# Rapid Assessment of Fish Freshness for Multiple Supply-Chain Nodes Using Multi-Mode Spectroscopy and Fusion-Based Artificial Intelligence

**DOI:** 10.3390/s23115149

**Published:** 2023-05-28

**Authors:** Hossein Kashani Zadeh, Mike Hardy, Mitchell Sueker, Yicong Li, Angelis Tzouchas, Nicholas MacKinnon, Gregory Bearman, Simon A. Haughey, Alireza Akhbardeh, Insuck Baek, Chansong Hwang, Jianwei Qin, Amanda M. Tabb, Rosalee S. Hellberg, Shereen Ismail, Hassan Reza, Fartash Vasefi, Moon Kim, Kouhyar Tavakolian, Christopher T. Elliott

**Affiliations:** 1 SafetySpect Inc., Grand Forks, ND 58202, USA; 2 Institute for Global Food Security, School of Biological Sciences, Queen’s University Belfast, Belfast BT9 5DL, UK; 3 Biomedical Engineering Program, University of North Dakota, Grand Forks, ND 58202, USA; 4 USDA-ARS Environmental Microbial and Food Safety Laboratory, Beltsville Agricultural Research Center, 10300 Baltimore Ave., Beltsville, MD 20705, USA; 5 Food Science Program, Schmid College of Science and Technology, Chapman University, Orange, CA 92866, USA; 6 School of Electrical Engineering and Computer Science, University of North Dakota, Grand Forks, ND 58202, USA; 7 School of Food Science and Technology, Faculty of Science and Technology, Thammasat University, Khong Luang 12120, Thailand

**Keywords:** fish freshness, food quality, shelf-life assessment, multi-mode spectroscopy, machine learning, artificial intelligence

## Abstract

This study is directed towards developing a fast, non-destructive, and easy-to-use handheld multimode spectroscopic system for fish quality assessment. We apply data fusion of visible near infra-red (VIS-NIR) and short wave infra-red (SWIR) reflectance and fluorescence (FL) spectroscopy data features to classify fish from fresh to spoiled condition. Farmed Atlantic and wild coho and chinook salmon and sablefish fillets were measured. Three hundred measurement points on each of four fillets were taken every two days over 14 days for a total of 8400 measurements for each spectral mode. Multiple machine learning techniques including principal component analysis, self-organized maps, linear and quadratic discriminant analyses, k-nearest neighbors, random forest, support vector machine, and linear regression, as well as ensemble and majority voting methods, were used to explore spectroscopy data measured on fillets and to train classification models to predict freshness. Our results show that multi-mode spectroscopy achieves 95% accuracy, improving the accuracies of the FL, VIS-NIR and SWIR single-mode spectroscopies by 26, 10 and 9%, respectively. We conclude that multi-mode spectroscopy and data fusion analysis has the potential to accurately assess freshness and predict shelf life for fish fillets and recommend this study be expanded to a larger number of species in the future.

## 1. Introduction

A surprising 30–40% of food is wasted every year [1], and seafood is not an exception [2]. Fresh seafood is highly perishable and can quickly deteriorate and lose quality.

Fresh fish products decay rapidly due to their biological composition, as well as physical and chemical characteristics such as the combination of high-water activity, neutral pH, low content of connectivity tissues, the presence of autolytic enzymes [3], and rising histamine content. Understanding the true remaining shelf life can conserve this important source of nutrition [4] and support decision-making regarding product sales and consumption or processing.

The ability to monitor seafood integrity at every possible node in the supply chain is of paramount importance for accountability while handling the seafood and ensuring value for the next node in the supply chain [5]. This study focuses on the distribution and retail stage of the supply chain, where the fish are in one of the following two forms: whole fish or fillet/portion/steak. For whole fish, trained personnel and even end users have access to all the external features providing multiple clues for sensory assessment of freshness. One can visually inspect the eyes, gills and skin and use smell and touch to estimate the remaining shelf life of the fish. A trained worker can distinguish the remaining shelf life of a whole fish within a half-day [6], but it is challenging to achieve such accuracy on fish fillets, the focus of this study.

Our research objective was to obtain an enlarged dataset permitting extensive and varied chemometric interrogation, including catabolites assay and conductance testing. To address the existing scientific gap, this study, for the first time, used multi-mode spectral measurements, including visible fluorescence, short-wave infrared (SWIR) reflectance, and visible-near infrared (VisNIR) reflectance to assess fish freshness. Classification methods analyzed the data from each of the spectroscopy modes individually, as well as using fusion methods that made use of all three spectroscopy modes. This multi-mode spectroscopy proof of concept, our hypothesis, potentially paves the way for a more comprehensive and extensive scan of the fish fillet tissue for freshness assessment.

### Background

**Figure 1:**
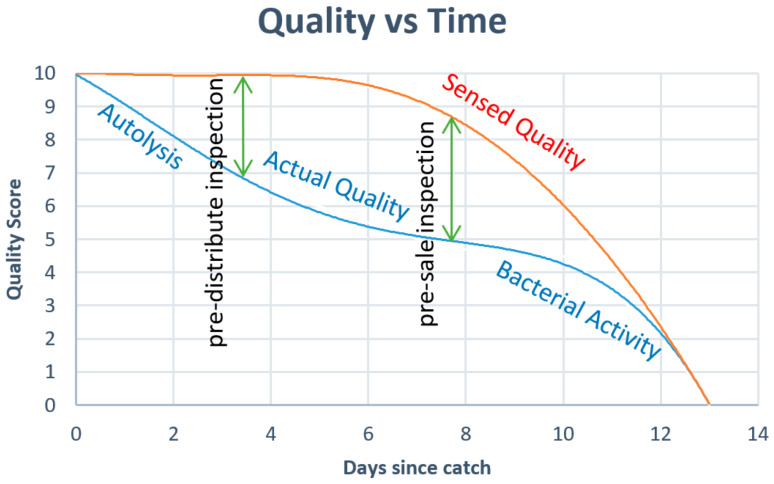
The inability of human sensing to detect decay at the molecular level in early stages [6].

The emphasis of this study is raw fish fillets, portions, and steaks. When the fish is filleted, it becomes more difficult to assess freshness even for experts [6]. The situation is exacerbated since by the time fish are filleted, they are in the hands of retailers, who may be less qualified to assess fish quality. Early fish decay is not easily detectable by human senses (Figure 1), causing fresh and partially degraded fillets to be treated equally. For seafood sold at retail, waste rates are 21.3% for fresh fish and 24.1% for fresh shellfish [7]. A technology capable of accurately and reliably estimating the remaining shelf life of fillets, using only the condition of the fillet, could lead to major waste reduction by allowing dynamic sales management. This includes sending fish with longer shelf life to more distant customers, marking down fish close to the end of their shelf life for faster sales, or sending them to secondary processing facilities where they can be converted to longer lasting products. A technology that automatically assesses the freshness of fish fillets can improve the prediction of the remaining shelf life, thereby reducing uncertainty in freshness assessment and creating an opportunity for waste prevention and revenue recovery.

The shelf life of fish is sensitive to numerous factors, including storage temperatures, packaging methods such as modified atmospheric, vacuum, and active and intelligent packaging [8]; whether and when the fish was eviscerated; method of catch; handling; and geographical origin. Tracking all these parameters for fish throughout the supply chain is challenging. Use of technology that can detect biological and chemical characteristics of fish tissue accurately and reliably in order to predict remaining shelf life could help to address shortcomings in information transfer through the supply chain.

There are several existing technologies to determine freshness of raw fish fillets: (1) Sensory methods, which use visuals for color and texture, as well as olfactory senses and touch to indicate firmness and water-binding capacity; (2) Total Volatile Base Nitrogen (TVB-N), which measures volatile gases like ammonia, dimethylamine and trimethylamine [9] and Gas sensors or electronic noses, which assess volatile compounds [10]; (3) Enzyme-linked Immunosorbent Assay (ELISA), which detects the presence of specific compounds using reactions between antigens and antibodies [11]; (4) Total Viable Count (TVC) microbiological analysis for determining specific spoilage organism (SSO) growth [12]; (5) Nucleotide/Adenosine Triphosphate (ATP) and K-value [13]; (6) Electric properties [14]; (7) Imaging which uses RGB photos to classify fish freshness; and (8) Fluorescence/reflectance spectroscopy [15].

There is a need for sensing techniques that allow for on-site detection of freshness in a rapid, cost-effective, and non-invasive manner [16]. Table 1 compares the different fish freshness assessment methodologies using the following criteria: (1) whether preparation work is needed, (2) test duration, (3) costs, (4) whether the method can be performed on-site, (5) whether there is a need for trained personnel, and (6) whether the test is destructive. Some of these methods, such as TVB-N, ELISA, TVC and Nucleotide assay, are more suitable for research, while others, like sensory and electrical properties, are applicable to industrial cases. However, each of these methods is associated with disadvantages, such as being expensive, time-consuming, requiring skilled operators, destructive to the fish, and/or associated with low accuracy.

Easy to use, handheld instruments have thus risen to prominence in recent years, promising portable analysis and the possibility of monitoring by personnel with basic training [17]. Currently, the industry state-of-the-art for portable fish freshness determination is potentiometric and is prescribed as suitable for batch monitoring but less reliable for measurements on individual fillets, which may be required by vendors selling to consumers at the endpoint of the food chain. Moreover, potentiometry is less accurate for skin-off fish fillets, which constitute a significant fraction of fillets for sale at retail.

These issues can be addressed by optical spectroscopic techniques. Recently, there has been significant work in spectroscopy and hyperspectral imaging of fish tissue [18,19]. Hyperspectral imaging allows collection of the full optical spectrum for every pixel of an image of a fish fillet [20]. Hassoun et al. [21] and Wu et al. [16] have reviewed current and emerging techniques in seafood freshness, including spectroscopic approaches, and more recently, Hassoun et al. [22] have highlighted the potential for spectroscopy in determining fresh versus thawed seafood.

Previously, Dufour et al. [23] studied freshness in four different fish species, including salmon, reporting changes in fluorescence spectral shape, but with limited spectral data and chemometric analysis. El Masry et al. [24] employed variable fluorescence excitation wavelengths and emission detection and discriminant analysis to differentiate between very fresh, fresh, and spoilt salmon fillets, attaining >80% accuracy in all cases. Similar studies have used fluorescence in combination with discriminant analysis and canonical correlation analysis (analogous to partial least squares) [25], as well as partial least squares regression (PLSR) [26].

Cheng et al. [20] used reflectance hyperspectral imaging, with wavelengths ~400–900 nm, to evaluate the K-value in grass carp and silver carp. Regression models were created from PLSR and a least square support vector machine (LS-SVM). The best results were obtained with an R^2^_p_ of 0.936. Khoshnoudi-Nia et al. [27] studied parameters within trout fillets, using reflectance hyperspectral imaging (430 to 1010 nm), with the best results of a value of 0.921 R^2^_p_ coming from LS-SVM which used six optimal wavelengths found by Genetic Algorithm (GA).

## 2. Materials and Methods

This study comprised two independent sets of experiments. Experiment set 1 (ES1) and associated analyses were conducted at the Institute for Global Food Security of the School of Biological Sciences at Queen’s University Belfast (QUB). Experiment set 2 (ES2) was performed at the USDA-ARS Environmental Microbial and Food Safety Laboratory, with analysis at the Biomedical Engineering Research Complex (BERC), University of North Dakota.

### 2.1. Materials

#### 2.1.1. Experiment 1: Farmed Atlantic Salmon

**Figure 2:**
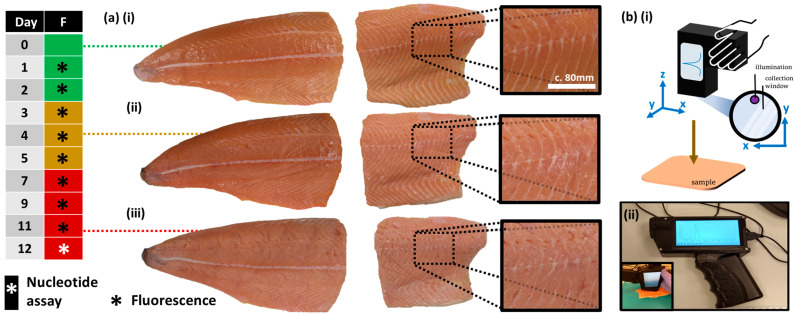
Experimental outline, samples, and apparatus. (**a**) Photographs of salmon fillet when (i) fresh, (ii) intermediate freshness, (iii) spoilt. Left: Freshness visual and olfactory assessment; green = fresh, amber = intermediate, red = spoilt. (**b**) Handheld fluorescence device (i) schematic and (ii) photographs.

**Figure 3:**
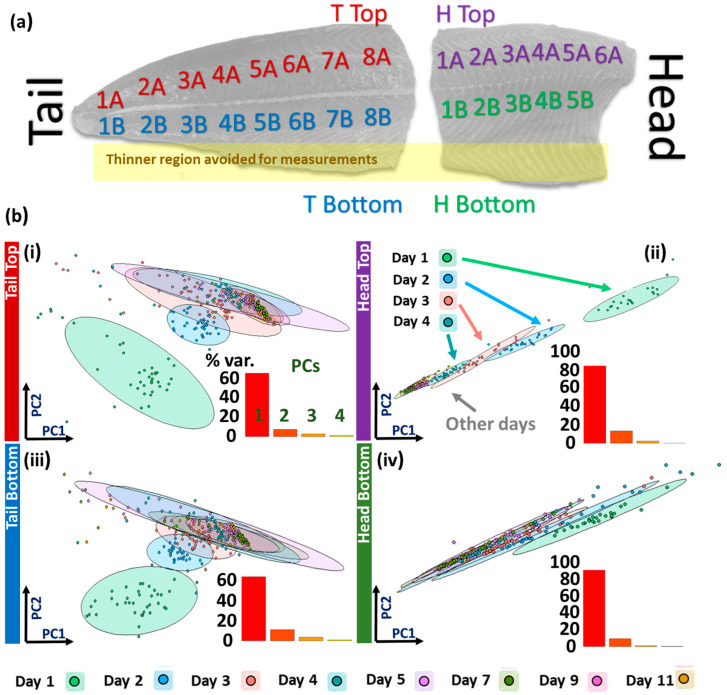
Location-specific PCA. (**a**) Salmon fillet with labelled measurement areas. (**b**) PCA score plots for (i) Tail Top, (ii) Head Top, (iii) Tail Bottom, (iv) Head Bottom regions. INSETS: PCA scree plots of percentage variance explained by each PC.

Two whole Atlantic salmon (*Salmo salar*) fillets were acquired from a fresh seafood store (Kilkeel, County Down, Northern Ireland), vacuum-packed, and transported to the laboratory at QUB in a polystyrene box with ice pack. The salmon were farmed and processed in Kilkeel fish factory. Age of fillets at point of purchase is up to three days post-mortem depending on when fish was harvested. The fillets were then labelled as ‘calibration’ and ‘validation’ samples and cut into two pieces each: ‘Head’ and ‘Tail’ sections for easy analysis (Figure 2 and Figure 3). Central sections were removed and placed into a freezer at −20 °C for an endpoint catabolites assay. The fillets were then placed in the fridge at 4–6 °C (39° F–43° F) and only removed for measurements (Supplementary figures).

#### 2.1.2. Experiment 2: Wild Coho Salmon, Wild Chinook Salmon, Sablefish

This dataset includes four fillets: one wild Coho Salmon (*Oncorhynchus kisutch*), one wild Chinook Salmon (*Oncorhynchus tshawytscha*), and two of Sablefish (*Anoplopoma fimbria*). These fillets were purchased from the Fulton Fish Market online store and were delivered frozen. Upon receipt they were placed in a −27 °C freezer and then put in a 4 °C refrigerator to thaw 24 h before day one of the experiment. Sample holders were 3D printed black thermoplastic. Plates of different thicknesses were also printed and placed in the holder under the fillet to ensure the distance between camera and fillet was the same for all samples. Plates were painted matte black on one surface to remove glare. Samples of 20 to 80 g were excised from fish before imaging and kept at −80 °C before being sent to Chapman University (Orange, CA, USA) for DNA-based species identification using methods described in [18].

### 2.2. Methods

#### 2.2.1. Experiment 1: Fluorescence Point Spectroscopy; Catabolites Assay

ES1 measurements included fluorescence point spectroscopy with a hand-held custom-made spectrometer, measurement of catabolites, and a skin dielectric measurement using the potentiometer Fish Freshness Meter (Fauldhouse, Scotland).

Fluorescence: ES1 measurements used a handheld, fluorescence spectrometer with 365 nm LED excitation, which while uncommon in the research literature, is more feasible for a hand-held commercial application, in contrast to spectroscopic devices within food studies at large [28,29]. Moreover, increased resolution is desirable, and therefore ES1 ran over 12 days where measurements were taken every day from day 1 to 5, inclusively, and every other day from day 7 to day 11, inclusively.

The fluorescence spectrometer and LED illumination exposure was set for an integration time varying between 5 and 400 ms to accommodate changes in fluorophore concentration as the fish fillet aged and the spectrometer gain was set to 100. Each spectrum was the average of 5 acquisitions. The fluorescence device incorporated a flat window to allow consistent orientation and distance to the sample surface. The wavelength resolution of the device was 2 nm.

Each fillet half-portion was assigned between 11 and 16 sub-areas for measurement depending on fillet size (1A, 1B, 2A, 2B, 3A etc.), where A denotes the dorsal (top) and B the ventral (bottom) portion of the fillet, and numbers increase from the posterior (tail) to the anterior (head) direction of the fillet (Figure 3). Five spatially random measurements were recorded per defined area. The probe window was wiped clean before moving to the next defined area. Measurements on the fillet lateral line and thinner peripheral fillet portions near the fillet bottom were avoided (Figure 3). In total, there were about 250 measurements across the two fillets (four half-fillet pieces) per measurement day and 2000 in total (1000/fillet), measured daily in a temperature-controlled laboratory.

A catabolites assay was performed (‘PRECICE^®^ Nucleotide Assay Kit, NovoCIB SAS, Lyon, France on Day 12 to confirm the calibration and validation fillets were of comparable freshness. As with other extraction assays, this process involves the cooking (c.100 °C) of fish portions (5–15 g) and blending (homogenizing) of the cooled fish tissue to release the catabolites. The samples were diluted with 1 mL of distilled water for each 1 g tissue. Homogenized samples were then filtered (180 μm thick; 11 μm pore size filter paper; Whatman^®^ grade 1). Three replicates were performed for each fillet. Fish filtrate extracts were centrifuged and pipetted into a well-plate, before enzymes were added to produce the reduced form of nicotinamide adenine dinucleotide (NADH), the absorbance of which serves as an indicator of respective catabolites concentration in the original fillet (NADH optical absorbance apex at 340 nm). The full assay procedure is available from NovoCIB SAS, Lyon, France. Freshness benchmarking was also performed using potentiometric measurements but no differences in freshness reading were noted. Later, the board of the potentiometer was found to be faulty. Therefore, potentiometer measurements are not presented in this paper. Visual and olfactory assessments were also conducted (Figure 2, left) according to an organoleptic chart (Distell). The cut-off for ‘rotten’ by Day 7 was supported by lack of change in spectral profile of the averaged spectra in Week 2. Measurements.

#### 2.2.2. Experiment 2: Line-Scan Spectroscopy in Fluorescence, VisNIR and SWIR Modes

In ES2 four fillets were each imaged six separate times, on every other day, with the fillets labelled as Day 1, Day 3, …, Day 11. The three hyperspectral imaging (I) modes consisted of fluorescence, SWIR, and VisNIR. Day 5 data was not used for the Sablefish samples, due to saturation within the fluorescence imaging mode on Day 5, rendering the images nearly useless. This issue was fixed for the later images by shortening the exposure time from 300 to 50 ms and scaling the data for exposure time.

Two different line-scan imaging systems, developed at USDA-ARS, were used to image the fillets [18]. The first system contained the capabilities for both fluorescence and VisNIR, while the second system acquired SWIR images. Both systems had a spatial resolution along instantaneous field of view (IFOV) of 0.4 mm/pixel and a line-scan incremental size of 0.4 mm.

For the first system, the VisNIR light source was a 150 W quartz tungsten lamp, and the fluorescence imaging light sources were two UV narrowband light sources with four 10 W, 365 nm LEDs each. The scan time for VisNIR and fluorescence were 1 m 20 s and 2 m 24 s, respectively. The VisNIHSI system imaged 125 wavelengths in the 419–1007 nm range, while fluoresIce HSI imaged 60 wavelengths in the 438–718 nm range. Scan number was 280. Therefore, the hyper cube size for VisNIR and fluorescence were 500 × 280 × 125 and 500 × 280 × 60, respectively.

For the second system, I SWIR HSI system had two lighting units, each housing four 150 W gold-coated halogen lamps with MR16 reflectors. The exposure time was 0.006 s. The scan time was 15 IThe SWIR HSI system imaged 287 wavelengths in the range of 842–2532 nm [18]. The scan number was 350. The hypercube size was 384 × 350 × 287.

HSI files were obtained from each system for each sample on each acquisition day, with additional white and dark images each day to allow spectral comparison that is independent of ambient light variation on any given day. Data conditioning methods are explained later in the analysis section for experiment 2.

## 3. Analysis

### 3.1. Experiment 1

Modelling: Principal component analysis (PCA) and an unsupervised self-organizing map (SOM) algorithm were conducted for data exploration (R Studio, Kohonen package) [30]. All PCA data was first mean-centered and scaled by the standard deviation i.e., standard normal variate (SNV) to produce a correlation matrix. The SOM algorithm calculated Euclidean distances, D, within and between nodes, containing the spectral vectors, a and b (Equation (1)).

(1) $$D=\sqrt{\sum_{i=1}^{n}{\left(a_{i}–\;b_{i}\right)}^{2}},\;$$

The SOM is a type of artificial neural network where multidimensional input data (vectors) are condensed into 2D space. In unsupervised mode, the technique may be considered as a non-linear generalization of PCA and is analogous to k-nearest neighbor (KNN) clustering analysis when performed in low-dimensional space. SOMs confer the benefit of maintaining topographical information: vectors proximal in n-dimensional space are also nearby in the SOM’s 2D representation. Although prominent in many different application spheres, SOMs have so far received comparatively little attention within food studies. Four different classification algorithms were employed: random forest (RF), support vector machine (SVM), linear discriminant analysis (LDA), and KNN clustering using the calibration fillet data, incorporating various pre-processing pipelines and five-fold internal cross-validation. Using data from the optimum Tail Bottom calibration fillet section, the ages of the fish were predicted from the equivalent section of the validation fillet (second fillet).

Fluorescence spectral features in the calibration fillet dataset, Peak 1 (P1) and Peak 2 (P2), were deconvolved where possible, using LabSpec5 software and Gaussian fits (Levenberg-Marquart method), peak ID, and an initial baseline subtraction. If the second fluorescence peak could not be identified, the spectral position of the inflexion point was noted and a suitable error ascribed. Cumulative error for the separation between P1 and P2, Δλ, was calculated additively. Fits were typically poor for a Gaussian function fit on fluorescence spectra; where available, P2 full-width half-maximum values ranged from 103.9–115.9 nm. The spectral position of P1 did not change significantly (452.6 nm ± 3 nm).

### 3.2. Experiment 2

#### 3.2.1. Data Pre-Processing

**Figure 4:**
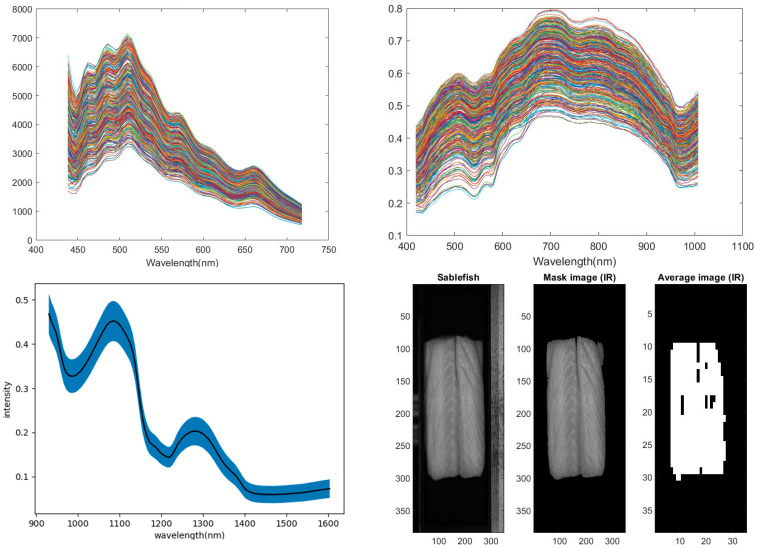
Sablefish training fillet (**top left**) all fluorescence spectra, (**top right**) all VisNIR spectra, (**bottom left**) SWIR mean spectra and one standard deviation range, (**bottom right**) the process of creating a mask and averaging the pixels to determine valid data.

The pre-processing code uses the hyperspectral images of the fish fillets from three different imaging modes as inputs. The imaging modes are reflectance in the VisNIR region, fluorescence in the visNIR region when excited at 365 nm, and reflectance in the SWIR region. A mask was created of the original images to remove all areas of the image that were not the fillet. After the spatial mask was created, the pixels in the masked image were resampled by spatial averaging over areas of 10 pixels by 10 pixels. This was done to extract measurements from the hyperspectral imaging system which would be comparable to the sampling area of the point spectroscopy measurements of Experiment 1 and also to prevent overfitting. The mean and standard deviation of the pixel intensities over the entire fillet were used to remove outliers in the data such as saturated pixels or deep grooves in the fish muscle. If greater than 10% of the pixels exceeded two standard deviations from the mean, for any wavelength band, the voxel was excluded from analysis. Graphs of spectra at each spatial coordinate, as well as masked and resampled images of the fillet were produced. Figure 4 shows spectra for fluorescence, VisNIR, and SWIR as well as a SWIR example of these images (at 1084 nm). The pre-processing was applied to all three spectroscopy modes. Mat files with the mean voxel spectra values shown in the spectral plot have been obtained for each imaging mode, and were used for the machine learning classifications.

#### 3.2.2. Classification

We used Python to implement a range of classification models. These were trained on a randomly selected set comprising 80% of the spectra from one fillet and tested on the remaining 20% of the spectra of that fillet. For Sablefish we had two fillets and Sablefish 1 was used for training, and Sablefish 2 was used for testing. The classification was first conducted using multiple base models to understand which perform the best. A wide variety of models were chosen, including decision tree, random forest (RF), Naive Bayes, k-nearest neighbor (KNN), linear discriminant analysis (LDA), support vector classification, logistic regression (LR), and a stacking ensemble method. These models were all implemented using functions from multiple libraries in Python. All of the models were used with their default settings, other than LR which was set to use a max iteration value of 1000 in order to maximize accuracy for the specific dataset.

**Figure 5:**
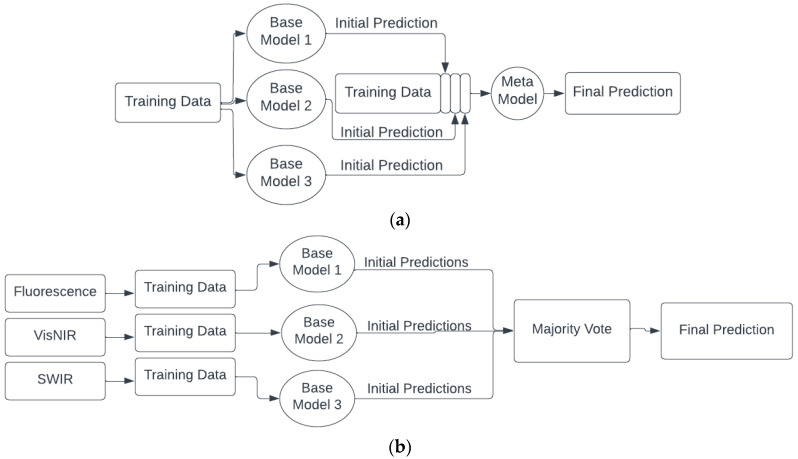

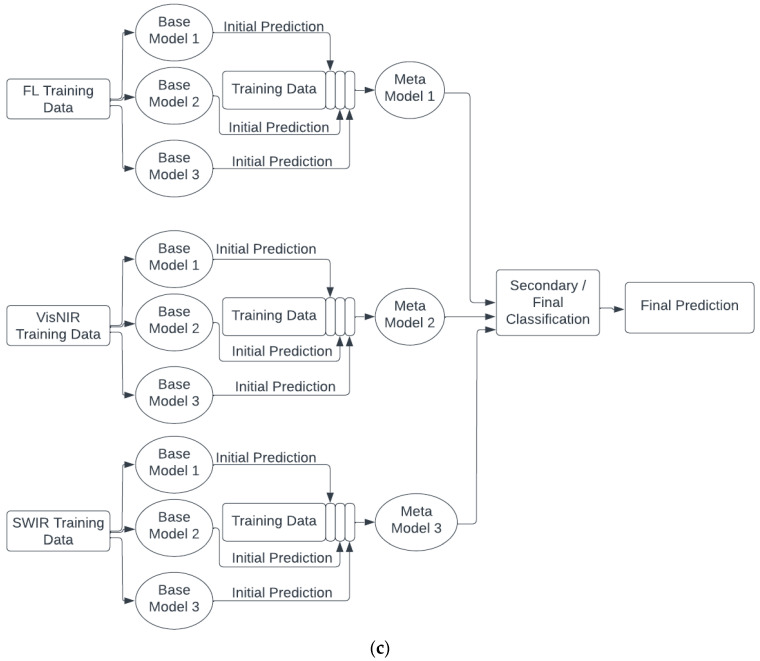
Different architectures for (**a**) single mode ensemble stacking, (**b**) decision level fusion with voting, and (**c**) decision level fusion with stacking and voting.

The stacking ensemble method used the three models trained on the dataset, LR, RF, and KNN as base models, because of their diversity in approach, then appended their predictions to the original dataset and used it to train a final meta model (LDA), which gave the final predictions (Figure 5). Considering multiple base models allowed for diversity and for each model’s predictions and errors to remain uncorrelated from each other. The meta model was also trained on a dataset of just the base models’ predictions, but this approach yielded up to 10% lower accuracy. LDA was chosen for the meta model because it had the highest classification accuracy when compared to the other models.

To use all three spectroscopy modes in the prediction of freshness, the decisions from all modes were entered into a voting system. This decision level fusion was implemented using an ensemble voting method. LDA and the stacking model both displayed very high accuracies over all three imaging modes, so decision level fusion was applied to optimize these models’ accuracies. When voting, the majority verdict was deemed as the final prediction. In the low probability event where the predictions from the three imaging modes happened to be different, the SWIR prediction would be used, as that was the mode that consistently garnered the highest accuracy. One should note that since two different devices measured the fillets (one for fluorescence and VisNIR and the other for SWIR), and the test voxels do not correspond to the exact same physical location on the fillet, reported fusion accuracies are at the level of the fillet as opposed to at the voxel level.

## 4. Results

### 4.1. Experiment 1

**Figure 6:**
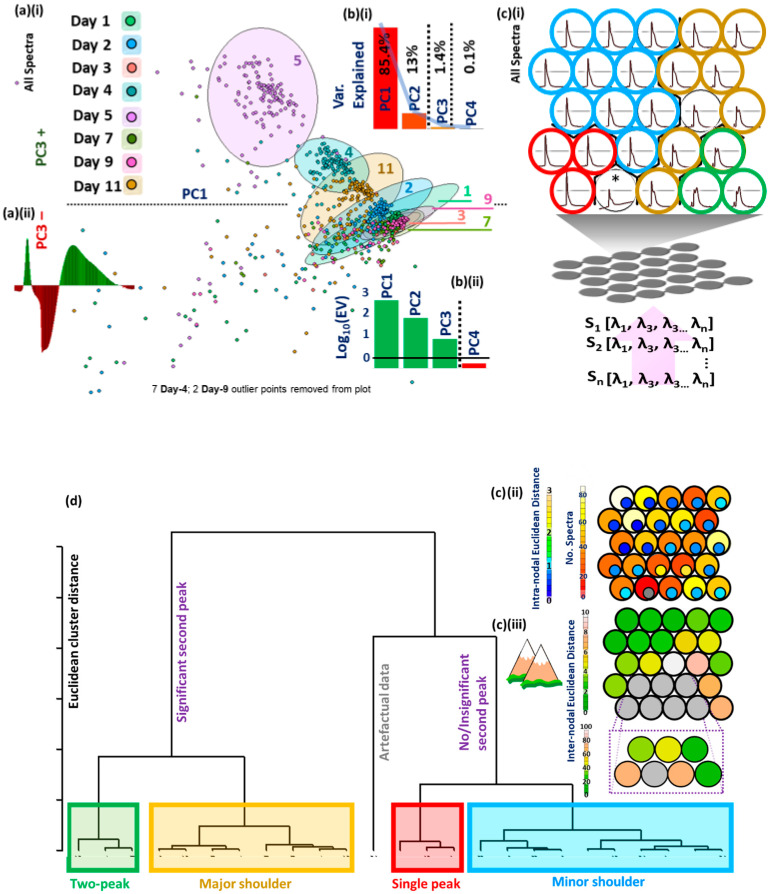
Exploratory data analysis. (**a**) (i) PCA plot for PC1 vs. PC3 (all spectra); (**a**) (ii) Eigenvalue plot for PC3. (**b**) (i) Scree plot for variance explained for PCs 1–4. Blue line shows inflection point between PC2 and PC3, or PC3 and PC4 (scree test) indicating either two or three PCs should be retained (dotted lines). (**b**) (ii) Log eigenvalue plot indicating that the first three PCs should be retained (eigenvalue > 1 test). (**c**) (i) Self organised map (SOM) codes plot with four categories identified by hierarchical cluster analysis (HCA): green circles (two-peak), yellow circles (major shoulder), red circles (single peak), blue circles (minor shoulder). (**c**) (ii) SOM heatmap with inset circles showing intra-nodal Euclidean distance. (**c**) (iii) Mountain plot showing inter-nodal Euclidean distance. (**d**) HCA dendrogram indicating clusters in (**c**) (i). * represents one anomalous spectrum that has been classified into its own node in the self-organizing map.

**Figure 7:**
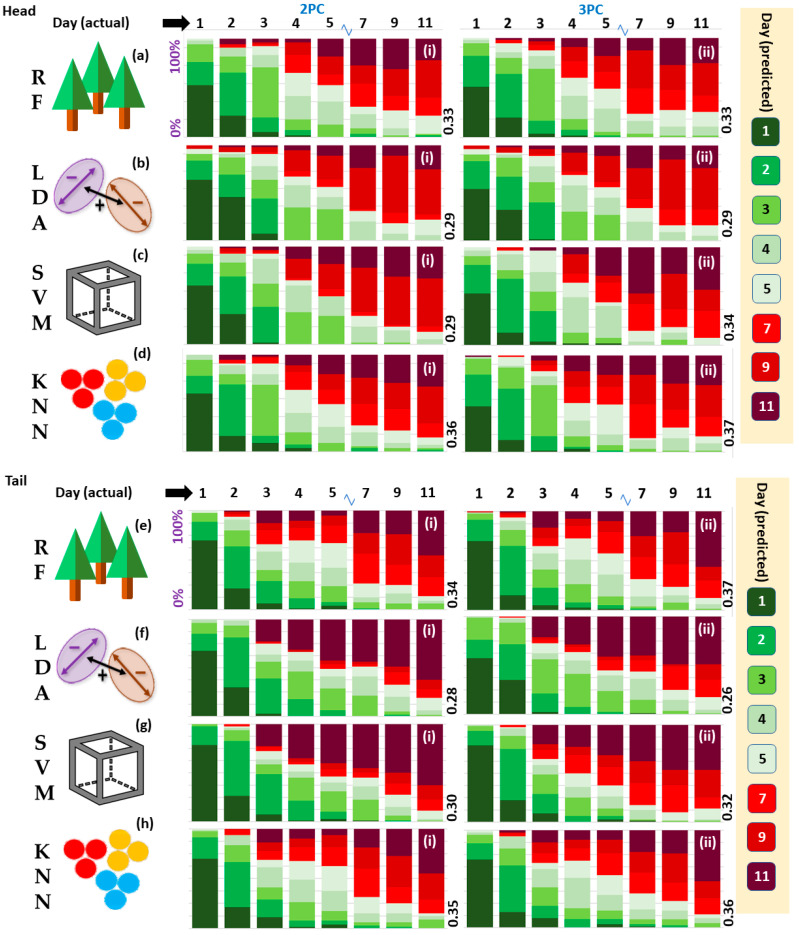
Five-fold cross-validation accuracies for Head models for (**a**) Random Forest, (**b**) Linear Discriminant Analysis, (**c**) Support Vector Machine, (**d**) K-Nearest Neighbour algorithms; (**e**–**h**) same for Tail models. Models built using 2PCs ((i) series), and 3PCs ((ii) series). Mean model accuracies given in white.

PC3 offers clearer class differentiation (Figure 6a) compared to PC1 and PC2. Interestingly, the loadings plot for PC3 displays clear negative correlation with the primary peak intensity (red bars, Figure 6a(ii)) and positive correlation with the emergence of the subsidiary fluorescence band (green bars, Figure 6a(ii)), the prominence of which is accentuated by the SNV pre-processing. A scree plot of variance explained by PCs is shown in Figure 7b(i) and a plot of log eigenvalues in Figure 7b(ii). We note the appearance of a ‘cut-off’ peak in the PC3 eigenvalue plot, which is probably due to the incomplete rejection of the fluorescence excitation band by the optical filter.

SOM analysis of all spectral data is displayed in Figure 6c(i). The SOM divides the data into four different class sets: 1. Two-peak (green circles); 2. Peak with significant subsidiary peak (yellow circles); 3. Peak with minor subsidiary peak (blue circles); 4. Single peak (red circles). The number of spectra allocated to each SOM node and the relative distance in space within a given node is presented in Figure 6c(ii). Distance between nodes is represented in Figure 6c(iii). Classes are categorized formally by hierarchical cluster analysis (HCA) in Figure 6d. The number of classes prescribed to HCA was set at four based on SOM evaluation. One artefactual spectrum was detected. It is not clear why the node in row 3, position 3 is so far in space from its neighbors other than some significant spectral profile differences between abutting ‘yellow class’ vectors and the effect of the pull from an anomalous spectrum in row 5, position 2. This also required the plotting of a rescaled outset.

Given the poor initial separation on all spectra, PCA was performed on both Head and Tail sections for both the Top and Bottom of the fillet (Figure 3a and Supplementary figures). Top and Bottom sectional analyses show similar 95% frequentist confidence ellipse (CE) patterns; Head and Tail sections display distinctly different CE patterns. Day 1 is well-separated in both Tail datasets. Partial separation is apparent for Days 2 and 3 (Figure 3b(i,iii)). Head datasets show much more oblately shaped 95% cEs for Top and Bottom fillet analyses (Figure 3b(ii,iv)); close to statistically significant separations for Days 1–4. (Insets) Scree plots indicate an increased coherence in the Head data, where PC1 explains over 80% of the variation, in contrast to closer to 60% in Tail data. Subsequent supervised models were thus built separately on Head and Tail datasets.

Replacing the original variables with reconstituted variables in the form of PCs confers the advantage of a more tractable computational model. Assuming that the PCs actually contain not only the most variation (true by definition), but also meaningful variation, which can be suspect in highly heterogeneous media, then a model built upon a small number of PCs reduces the possibility of overfitting and the inability of the analytical model to generalize to unseen data [31].

PC retention was decided based upon a visual scree test, where the inflexion point occurs at the point of PC2 or PC3. This criterion holds for scree plots based on PCA analysis of all spectra or indeed those related to Head or Tail only models. Similarly, eigenvalue > 1 criterion (EOC) prescribes retention of the first three PCs (Figure 6b(ii)) for all spectra [32]. This requisite entails that any retained PCs must explain more variation than that explained by the original variables i.e., the eigenvectors, λ, must be ‘stretched’ to account for greater variation in n-dimensional space (Equation (2)). Cumulative Percentage Variation (CPV) explained, normally set arbitrarily at 95%, was not considered. This criterion would entail the retention of many redundant PCs from the Tail model. Models were created for Savitzky-Golay smoothed spectra and unsmoothed spectra; no classification differences were observed, therefore all spectra presented herein are unsmoothed.

(2) $$\%Variation=\frac{\lambda_{i}}{\sum_{i=1}^{n}\lambda_{i}},\;$$

Five-fold cross-validation (CV) was performed to assess the performance of four different ML algorithms on the salmon datasets (Figure 7). The mean individual model classification accuracies are presented in Figure 7. The mean performances for RF, LDA, SVM, and k-NN models were 34.3%, 28.1%, 31.4%, and 36.1%, respectively. Head and Tail models returned similar mean accuracies (32.6%, 32.4%) while models built on PCs 1, 2, and 3 outperformed those built on PC 1 and PC 2 only (33.2%, 31.7%). The best classification accuracy was returned by a Tail RF model with 3 PCs (37.0%). We think a limitation of this study is the small number of data point samples which were significantly increased in the second experiment.

**Figure 8:**
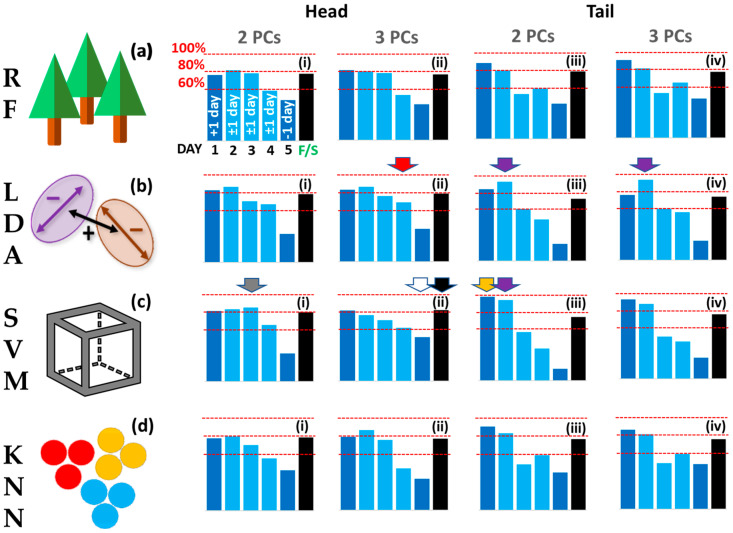
Model classification accuracies ±1 day for (**a**) Random Forest, (**b**) Linear Discriminant Analysis, (**c**) Support Vector Machine, (**d**) K-Nearest Neighbour algorithms for models Head 2PC ((i) series), Head 3PC ((ii) series), Tail 2PC ((iii) series, Tail 3PC ((iv) series). Accuracies for Days 2, 3, and 4 (light blue bars) are ±1 day; Day 1 is +1 day only (Day 2); Day 5 is −1 day only (Day 4). Black bars: fresh vs. spoilt accuracy where ‘fresh’ is Days 1–5 and ‘spoilt’ is Days 7, 9, 11. Coloured arrows represent best classification accuracies: Day 1, yellow arrow; Day 2, purple arrows; Day 3, grey arrow; Day 4, red arrows; Day 5, white arrow; Fresh vs. Spoilt, black arrow.

**Figure 9:**
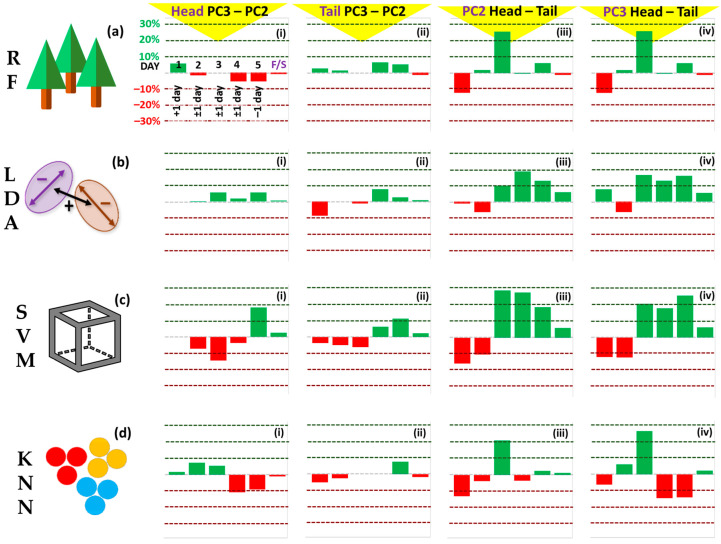
Differential accuracy plots derived from data in Figure 8 for (**a**) Random Forest, (**b**) Linear Discriminant Analysis, (**c**) Support Vector Machine, (**d**) K-Nearest Neighbour algorithms for models Head 3 PCs—Head 2 PCs ((i) series), Tail 3 PCs—Tail 2 PCs ((ii) series), 2 PCs Head—2 PCs Tail ((iii) series), 3 PCs Head—2 PCs Tail ((iv) series).

Accuracy was also surveyed for time spans larger than one day as shown in Figure 8. In the second case we used +/− one day, so effectively a 2 or 3 day window, and in the third case, we grouped the fish as fresh (days 1–5) or spoilt (days 7–11). Optimal classifications for Day 1 (yellow arrow), Day 2 (purple arrows), Day 3 (grey arrow), Day 4 (red arrow), and Day 5 (white arrow) are also pointed out in Figure 8. The models were assessed for discrimination between ‘fresh’, prescribed as Days 1–5, and ‘spoilt’, prescribed as Days, 7, 9, and 11, samples (black bars, Figure 8). Here, all models performed equitably (c.80%). The best performance is noted for the Head SVM 3PCs model (82.5%) (black arrow, Figure 8c(ii)). Differentials are plotted in Figure 9 to accentuate the differences between PCs used (Figure 9 (i series, ii series)) and Head and Tail models (Figure 9 (iii series, iv series) for the four algorithms (a)–(d).

**Figure 10:**
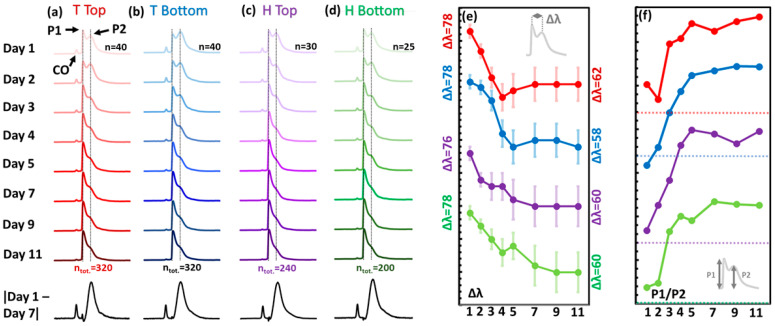
Mean spectra (SNV-scaled) for (**a**) Tail Top, (**b**) Tail Bottom, (**c**) Head Top, (**d**) Head Bottom fillet sections. Bottom (black spectra): Differential modulus spectra for Day 1 (fresh) minus Day 7 (spolit). (**e**) Spectral wavelength separation between P1 and P2 (nm); offset for clarity. Outset Δλ values are initial and endpoint peak separation values. *Y*-axis division = 2 nm (**f**) Intensity ratio P1/P2; offset for clarity. Dotted lines = unity position. *Y*-axis division = 0.1. Labelled in (**a**): P1 = Peak 1, P2 = Peak 2, CO = Cut-Off Peak.

**Figure 11:**
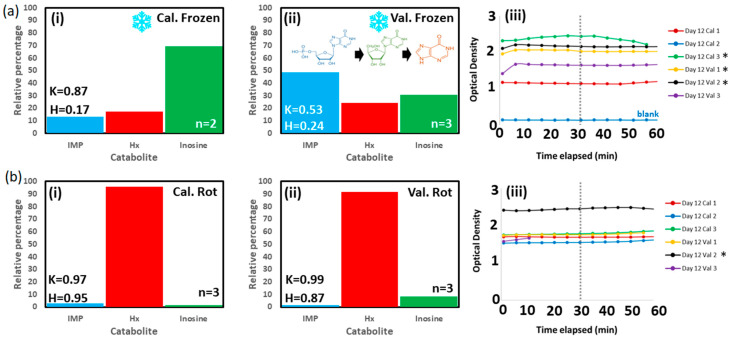
(**a**) Day 12 measured catabolites profiles of frozen (on Day 0) for (i) calibration and (ii) validation fillets. INSET catabolic pathway: Inosine Monophosphate->Inosine->Hypoxanthine. (iii) UV absorption kinetics. (**b**) Same for rotting (i) calibration and (ii) validation fillets refrigerated but not frozen throughout experiment. (iii) Rotting salmon absorption kinetics. INSET K-values, H-values, and number of replicate measurements averaged (*n*). Optical density read at 30 min as prescribed. * The optical absorbance measurement in the plate reader has been assumed to be linear.

A clear trend of decreasing peak separation, Δλ, and increasing P1/P2 ratio was displayed as the fillet aged. Figure 10 shows mean spectral profiles and accompanying peak separation (P1->P2, Δλ, in nm) and relative peak intensity ratios (P1/P2) for the four salmon fillet regions for Days 1–11. Δλ was most marked for the Tail Bottom section at 20 nm (blue line, Figure 10e). Similarly, Tail Bottom was adjudged to provide the best model considering P1/P2 intensity ratio, returning a monotonic increase from Days 1–9. Tail Bottom data was used to test the validation salmon fillet, which showed freshness scores comparable to the calibration salmon fillet for both Day 0 and Day 12. This was based on catabolites assay analysis (Figure 11), where the relative percentage of three catabolites, inosine monophosphate (*IMP*), inosine, and hypoxanthine (*Hx*) are compared. Freshness is quantified by using the well-known, simplified *K*-value and alternative *H*-value metric (Equation (3)) [33], which considers the relative catabolites composition effect on bitter taste [34]:

(3) $$K=\frac{Inosine\times Hx}{Inosine\times IMP\times Hx},\;\;H=\frac{Hx}{Inosine\times IMP\times Hx},\;$$

**Figure 12:**
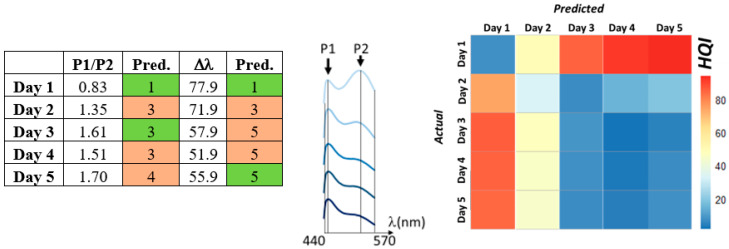
ES1 (**left**) Predicted freshness day of validation salmon dataset (Tail, Bottom) based on P1/P2 ratio peak intensity (third column), and on P1–P2 peak separation (Δλ, fifth column). Comparisons with Tail Bottom calibration data in Figure 10. (**Middle**) Validation spectra, Days 1–5, 440–570 nm truncated. (**right**) Goodness of fit test for validation dataset (Tail, Bottom; 440–570 nm truncated). Hit Quality Index (HQI) scale shows how well validation spectra conforms to equivalent calibration spectra where lower values (blue) represent better fits and higher values (red), worse fits.

Models based on Δλ and P1/P2 correctly predicted the trend for decay but were often ±1 day (Figure 12a). Similarly, a goodness-of-fit test, predicted the overall trend correctly but proved imprecise in terms of exact freshness day (Figure 12b). This test summed the absolute values of the difference between scaled calibration fillet and validation fillet spectral datapoints, *x*, and *y*, where a lower value of the Hit Quality Index (𝜒) indicates a better fit (Equation (4)).

(4) $$\chi =\sum_{i}^{n}\left|x_{i}-y_{i}\right|$$

### 4.2. Experiment 2

Results were obtained for all four fillets from three species (Coho Salmon, Chinook Salmon and Sablefish). In addition, for Sablefish one fillet was used for training and the other for testing. In general, the highest scoring single mode models for each species were the discriminant analyses (LDA/QDA) and stacking, using the SWIR spectroscopy mode. The accuracies for each spectroscopy mode are shown in Table 2, alongside the accuracy of the decision level fusion (Table 3). Both testing and training accuracies are displayed to assist in demonstrating possible overfitting. The confusion matrix of voxels for Sablefish using decision level fusion and stacking tested on an unseen fillet is shown in Table 4.

The discriminant analyses and stacking consistently performed the best amongst each species. LDA, QDA and stacking also obtained the highest accuracies of any single mode model when testing on one sample of Sablefish and being trained on the other.

The fusion method improved the accuracies drastically. The accuracies obtained by the fusion approaches for each species trained and tested on themselves were above 99% when each class corresponds to the fillet’s freshness within +/− one day.

When classifying freshness, within +/− one day, by training on one sample of Sablefish and being tested on the other, the decision level fusion using LDA achieved an accuracy of 95%, nearly a 9% increase in accuracy from the single mode approach obtained by LDA SWIR. The fusion method with the previously mentioned models appears to be the best overall method for the intended use, which is training on different samples than are being tested.

The confusion matrix for the case of training with one fillet of sablefish and testing with the other using decision level fusion and LDA is shown in Table 4. Each cell shows the number of voxels predicted as a certain day and the true post-mortem duration.

Further increases in accuracy could be obtained by taking multiple measurements and averaging the predictions or taking a majority vote to gather a final prediction. Taking three measurements at 95% each would equate to an overall accuracy of over 99% in the case of Sablefish (Supplementary figures). The capability to improve freshness assessment performance is unique to spectroscopy and does not apply to other lab techniques such as catabolites assay, TVBN, etc.

## 5. Discussion

### 5.1. Experiment 1

In our study, while improved separation is found in PC3 in the all-spectra analysis, the lack of class separation in the PC score plot on all spectra resulted in dividing the data into separate location-specific models. Subsequently, models were built using separated Head data and Tail data given that Top and Bottom fillet sections proved similar in appearance of confidence ellipse (CE) shape position in the PC scores plot. Moreover, the clear spatial progression of the CE positions in the separated Head and Tail suggests genuine biological/chemical change rather than artefactual data or factors exogenous otherwise.

The SOM analysis permits unambiguous identification of spectral types and tractable hierarchical cluster analysis (Figure 6d). The top left nodal landscape is not only well-populated (yellow, light yellow nodes, heatmap in Figure 6c(ii)) but also comparatively homogeneous i.e., close proximity in n-dimensional space, in terms of intra-nodal separation (dark blue enclosed circles, Figure 7c(ii)) and inter-nodal separation (dark green nodes, Figure 6c(iii)). Moreover, very fine, single peak spectra are not observed in average spectral plots, but the trend in Figure 10 suggests that they become more common as the fillet ages. This suggests that even where spectra show clearly separated multiple fluorescence peaks, there is still clear inter-class variance and significant averaging may be necessary for sufficiently accurate freshness day classification.

The Tail SVM 2PC model achieves ne”r-perfect classification ±1 day for Days 1 and 2 (97.5%, 93.8%), but returns a significant drop-off in accuracy for Days 3 and 4 (56.3%, 37.5%). The comparative inaccuracy in the multiclass identification lies in significant intra-class variation. Thus, multiple measurements must be taken and evaluated.

The study has a number of limitations. The function of the catabolites assay is to gauge the equivalence, or lack thereof, of freshness condition of the fillets. The assay purpose is not to give an exact indication of the days post-mortem; to do so, proper calibration would need to be performed, likely considering the exact type of salmon, yearly season, and exact fillet region from which the tissue was taken. We note that while the fillets are of similar freshness on Day 0, there is nevertheless some discrepancy (Figure 11a(i) vs. Figure 11a(ii)). Based on our own preliminary catabolites measurements, we estimate this difference as approximately one day (i.e., calibration fillet is one day fresher). This correction could, in turn, increase the accuracy of prediction in Figure 12, however, a clear difference remains in spoilage rate predicted.

The PC variance profiles, as identified in the scree plots, are notably different for Head and Tail data where Tail datasets are much more spread out amongst PCs with PC1 accounting for merely 60% of the variation (Head PC > 80%). This suggests that the dataset may contain significant amounts of prominent redundant information, intra-class variation, which contributes to the cardinal PCs and reduces model accuracy. Thus, while parsimony in the form of dimensionality reduction makes classification models more manageable, this does not always constitute better model performance.

### 5.2. Experiment 2

In real world applications, the test fillet is totally unseen, therefore among the three fish species studied in this research, Sablefish would offer the closest accuracies resembling what would be seen in practice. This is due to Sablefish having two fillets to sample, allowing one fillet to be trained on, while the other is tested, providing a more objective measure of the accuracy of our model. Therefore, the objective accuracy is 95% for predicting within +/− one day using decision level fusion and stacking.

The accuracy of multi-mode spectroscopy along with fusion of the modes can be improved by taking several measurements and relying on the majority vote. Measuring different points on the fillet using multi-mode spectroscopy does not impose any cost on the user and takes a few seconds each. Therefore, the nature of multi-mode spectroscopy allows for improving the accuracies without incurring cost.

For sablefish trained and tested on different fillets, the accuracy within +/− one day, which is 95%, increases to 96% if fish is categorized into three freshness grades (fresh, fairly fresh, and spoilt) and to 100% if fish is categorized into two freshness grades (fresh and spoilt).

Careful examination of the confusion matrix for the two Sablefish fillets with decision level fusion and LDA (Table 4) shows that the cells away from the diagonal of the confusion matrix are mostly zero. This shows that even if a voxel is misclassified, the predicted class is not randomly distributed among the other classes but closer to the true day number.

Our results show that multi-mode spectroscopy achieves 95% accuracy, improving the accuracies of the FL, VIS-NIR and SWIR single-mode spectroscopies by 26, 10, and 9%, respectively.

Overall, considering both ES1 and ES2, the accuracy of 95% obtained in this study for the prediction of freshness in Sablefish fillets within +/− one day using multi-mode spectroscopy cannot be compared directly to the literature, because, to the authors’ best knowledge, fusion of fluorescence, VisNIR, and SWIR has been performed in this study for the first time. Therefore, the fusion results will be compared separately, with single mode fluorescence and reflectance spectroscopy.

For fluorescence, Karoui [25] obtained the highest accuracy in literature with 91.67%. This is lower than the fusion accuracy of 95% reached in this study. For reflectance, the two studies in literature with the highest accuracies, by Cheng [20] and Khoshnoudi-Nia [27], both performed regression. Since our results are based on classification and not regression, using our confusion matrix, our classification accuracy has been translated to an R2 of 0.983. This value exceeds the R^2^ of 0.936 and 0.921 reported by Cheng [20] and Khoshnoudi-Nia [27], respectively, demonstrating that multi-mode spectroscopy outperforms single mode reflectance spectroscopy.

## 6. Conclusions

In this study, fluorescence, VisNIR, and SWIR spectroscopy, as well as the fusion of the three, have been studied, and multi-mode spectroscopy was proven to be a tool superior to single mode spectroscopy in obtaining high accuracies in fish freshness classification. The machine learning methods used to fuse different modes displayed their ability to improve upon what single mode spectroscopy could not achieve even with deep and detailed data visualization. Multimodal fusion techniques improved accuracies when compared to classifying on only single mode data even when, for single mode, the fillet is divided to specific regions with specific chemometric characteristics.

The highest performances from single mode fluorescence, VisNIR and SWIR spectroscopy were 69% with stacking, and 86% and 85% with LDA, respectively. When the three modes were combined, the accuracy increased to 95% with LDA. Stacking accuracies were generally only 1% lower than LDA.

## 7. Future Work

In this research, ES2 has been conducted as a proof of concept to understand the level of accuracy that multi-mode spectroscopy can achieve in a lab setting. The future work from this research group will involve developing a handheld device capable of measuring all three spectroscopy modes in a single point measurement. In the next step, to move towards commercialization, all three modes will be measured all at once in a multi-mode point spectroscopy system with its own illumination and AI chip that can be used in processing facilities, distribution centers, grocery stores, and restaurants.

Additional studies can also be conducted on larger datasets that include more species, as well as multiple samples within the same species to enable training on one sample, while testing on the others. This approach would better resemble the process that would occur in the field. Also, future work can combine fish species identification algorithms developed in our group [18,19,35] with freshness classification and to consider other quality features of fish including detection of histamine in fish fillets.

This study can be expanded by incorporating transformative technologies such as blockchain that can enhance the capabilities of seafood supply chain systems. More precisely, we are working on the integration of blockchain technology with Internet of Things (IoT)-enabled devices such as our spectroscopic handheld devices [36]. The resilient features of blockchain technology include immutability, decentralization, verifiability, and trust. These features combined with the intelligent capability provided by AI technology will help to reduce food waste through the detection of fish freshness and estimation of shelf life as well as prevent food frauds, such as adulteration and its related risks, hurting food supply chain systems.

## Figures and Tables

**Table 1:** Comparison of fish freshness assessment methods. TVBN: Total Volatile Basic Nitrogen, ELISA: Enzyme-linked Immunosorbent Assay, TVC: Total Viable Count, ATP: Adenosine Triphosphate, Lab Equip Sal: laboratory, equipment and salary. All methods apply to fish fillets except for Electric Properties and RGB Imaging that work on whole fish. Green, orange and red represent the three levels from the most to the least desirable.

	Method	Prep	Duration	Cost	In-Situ	Skilled Worker	Destructive	Applicable to Fillets
1	Sensory	no	seconds	salary	yes	yes	no	yes
2	TVBN	yes	hours	lab, equip, sal	no	yes	yes	yes
3	ELISA	yes	hours	lab, equip, sal	no	yes	yes	yes
4	TVC	yes	hours	lab, equip, sal	no	yes	yes	yes
5	Nucleotide/ATP	yes	hours	lab, equip, sal	no	yes	yes	yes
6	Electric Properties	no	seconds	device	yes	no	no	no
7	RGB Imaging	no	seconds	device	yes	no	no	no
8	Spectroscopy	no	seconds	device	yes	no	no	yes

**Table 2:** Accuracies for single mode, Color code accuracies: green > 95%, 83% < yellow < 95%, red < 83%.

**Classes Day 1,3,7,9,11**	**Sablefish#1**						**Sablefish #2**						**Sablefish #1 Train, Sablefish#2 Test**					
**Single Mode**	**FL**		**SWIR**		**VisNIR**		**FL**		**SWIR**		**VisNIR**		**FL**		**SWIR**		**VisNIR**	
**Train/Test**	**Train**	**Test**	**Train**	**Test**	**Train**	**Test**	**Train**	**Test**	**Train**	**Test**	**Train**	**Test**	**Train**	**Test**	**Train**	**Test**	**Train**	**Test**
RF	100	71	100	52	100	60	100	71	100	59	100	72	100	50	100	30	100	38
KNN	77	67	63	44	69	49	80	68	66	53	78	65	80	52	68	29	79	34
LR	96	93	35	36	59	56	93	91	39	42	65	66	92	69	41	30	64	38
LDA	93	91	100	99	98	95	91	88	100	99	98	96	91	66	99	86	98	85
QDA	100	96	100	96	100	88	100	88	100	95	100	82	99	65	100	78	100	68
Stacking	97	94	100	100	99	96	95	91	100	99	99	96	94	69	99	85	98	84
**Classes: Day 1,3,7,9,11**	**Coho Salmon**									**Chinook Salmon**								
**Single Mode**	**FL**			**SWIR**			**VisNIR**			**FL**			**SWIR**			**VisNIR**		
**Train/Test**	**Train**	**Test**		**Train**	**Test**		**Train**	**Test**		**Train**	**Test**		**Train**	**Test**		**Train**	**Test**	
RF	100	94		100	47		100	55		100	70		100	52		100	57	
KNN	97	96		63	39		71	50		84	70		63	41		67	44	
LR	100	98		38	34		45	48		94	92		29	27		40	40	
LDA	96	97		100	100		94	91		87	86		100	100		98	96	
QDA	100	98		100	99		100	79		100	91		100	97		100	70	
Stacking	100	99		100	100		99	94		96	94		100	100		99	95	

**Table 3:** Accuracies for decision-level fusion and stacking. Color code accuracies: green > 95%, 83% < yellow < 95%, red < 83%.

Classes: Day 1,3,7,9,11	Coho Salmon						Chinook Salmon					
Single Mode	FL		SWIR		VisNIR		FL		SWIR		VisNIR	
Train/Test	Train	Test	Train	Test	Train	Test	Train	Test	Train	Test	Train	Test
RF	100	94	100	47	100	55	100	70	100	52	100	57
KNN	97	96	63	39	71	50	84	70	63	41	67	44
LR	100	98	38	34	45	48	94	92	29	27	40	40
LDA	96	97	100	100	94	91	87	86	100	100	98	96
QDA	100	98	100	99	100	79	100	91	100	97	100	70
Stacking	100	99	100	100	99	94	96	94	100	100	99	95

**Table 4:** Confusion matrix of voxels for Sablefish using feature level fusion and LDA tested on an unseen fillet. Color code accuracies: green > 95%, 83% < yellow < 95%, red < 83%.

		Predicted Day				
		1	3	7	9	11
True Day	1	378	0	0	0	0
	3	0	368	0	0	0
	7	0	0	343	9	0
	9	0	0	65	249	9
	11	0	0	2	10	308

## Data Availability

Data available on request due to privacy restrictions.

## Supplementary Materials

The following supporting information can be downloaded at: https://www.mdpi.com/article/10.3390/s23115149/s1, Figure S1. Cumulative time spent outside of refrigeration for calibration and validation salmon fillets; Figure S2. Location-specific PCA analysis for calibration salmon fillet (Day 1). INSET: fillet locations and discoloration of Tail section end (pink box). C = Calibration fillet, H= Head, T = Tail. * = regions near fillet periphery that returned highly localized, partially separated confidence ellipses. Confidence ellipses are at 95%; Figure S3. Selected PC1 vs. PC2 location-specific PCA plots for Days 2–5. (a) Day 2, (b) Day 3, (c) Day 4, (d) Day 5. 95% confidence ellipses;. Figure S4. Additional location-specific PCA plots for Day 1. (a) PC3 vs. PC4, (b) PC5 vs. PC6, (c) PC7 vs. PC8, (d) PC9 vs. PC10. 95% confidence ellipses; Figure S5. Increase in accuracy by measuring multiple points on the test sablefish fillet and how fusion can decrease the number of measurements to achieve the same accuracy level.
