# Supplementary material for: Rapid Assessment of Fish Freshness for Multiple Supply-Chain Nodes Using Multi-Mode Spectroscopy and Fusion-Based Artificial Intelligence
Source: Sensors (Basel). 2023 May 28;23(11):5149. doi: 10.3390/s23115149 (PMC10255221; doi:10.3390/s23115149)
Supplement: Supplementary file 1 [file sensors-23-05149-s001.zip › sensors-2266005-SI.pdf]

–Supplemental Information–

Both the calibration and validation salmon fillets spent the same amount of time outside of refrigeration during the experiment (Supplementary Figure S1). The precise age of each sample post-mortem at time of purchase is ±3 days. The relative difference in age is estimated to be approximately one day based on preliminary catabolites assays in the QUB ASSET lab and catabolites data in Figure 12 (main text).

The prominence of intra- and inter-class variance was studied by looking at PCA plots for different spatial regions within given days up to PC10 (Supplementary Figures S2–S4). Most analyses were agnostic to measurement location, demonstrating the comparative similarity of measurement location on the fillet when compared to differences between days. An exception was the peripheral regions of the Tail section which showed separation from most other regional clusters (Supplementary Figure S2), possibly due to scattering effects from the proximal substrate interface (thinner flesh). Further, some of the most peripheral tail regions (Tail 1A, 1B) have clear discoloration indicating compositional difference and possible damage during handling on the boat or during processing (Inset, Supplementary Figure S2).

The Torrymeter potentiometric device works by passing a small current through the fish tissue and recording the resulting phase discrepancy between voltage and current sine waves caused by the capacitive reactance action of the cell membrane. Measurements taken using the 'TORRY STD' setting. 10 sets of 10 measurements were recorded on the skin side, upper shoulder, for the head portions of the calibration and validation fillets, as prescribed in the Torrymeter guidelines. The device was kept in position for each set of 10 measurements, after which it was removed, the head electrodes cleaned, and another set of 10 acquired on a proximal upper shoulder region. Torrymeter measurements were conducted on Day 0 as a baseline and then from Day 3 to the experiment conclusion on Day 12 to ascertain the point of spoilage i.e. when the fillet would no longer be considered acceptable for human consumption. The potentiometric data failed to discriminate between fresh and rotting salmon fillet. Later the device's board was found to be faulty. Therefore, Torrymeter measurements are not presented in this article.

Sensory measurements were similarly conducted from Day 3 until Day 13 and descriptors on the Distell organoleptic charts consulted. Based on this, Days 0, 1 and 2 were adjudged fresh, Days 3, 4 and 5, intermediate, and Days 7, 9 and 11 spoilt. For the purpose of binary classification, Days 0–5 were adjudged fresh, and Days 7, 9, and 11, spoilt.

Gaussian fits were poor owing to the nature of the fluorescence phenomenon: incident photons are absorbed by a molecule causing excitation to a vibrational level in the first excited electronic energy state before cascading back to the ground state via the various vibrational energy levels associated with the first excited and ground electronic states, emitting photons of varying energies, and resulting in a broad spectral profile.

Most Day 1 95% (frequentist) confidence ellipses in higher PCs (all spectra) overlap (Supplementary Figure S2), meaning no significant differences are present. Measurement region 8 could be removed, which shows some highly localized data that is statistically separated from most other clusters. A similar conclusion is reached in the study of PCs for days 2 to 5 (Supplementary Figure S3; selected plots) and the study of higher PCs for Day 1 (Supplementary Figure S4).

Supplementary Figure S5 shows how the performance of decision level fusion with stacking can improve by repeating the sampling the test fillet.

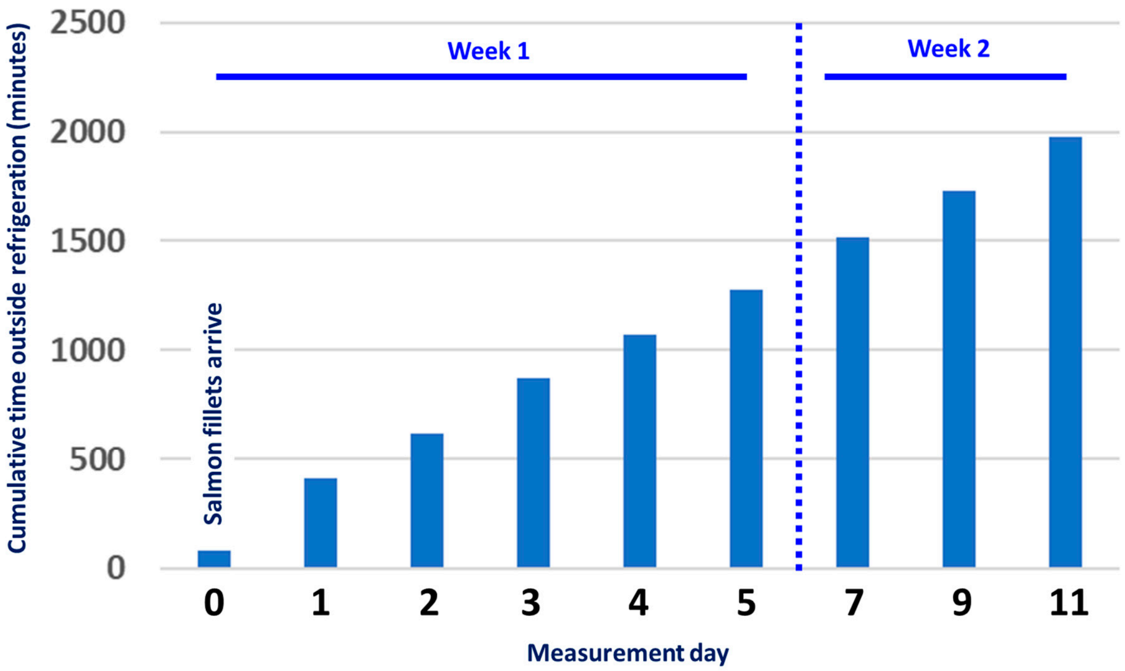

Supplementary Figure S1. Cumulative time spent outside of refrigeration for calibration and validation salmon fillets.

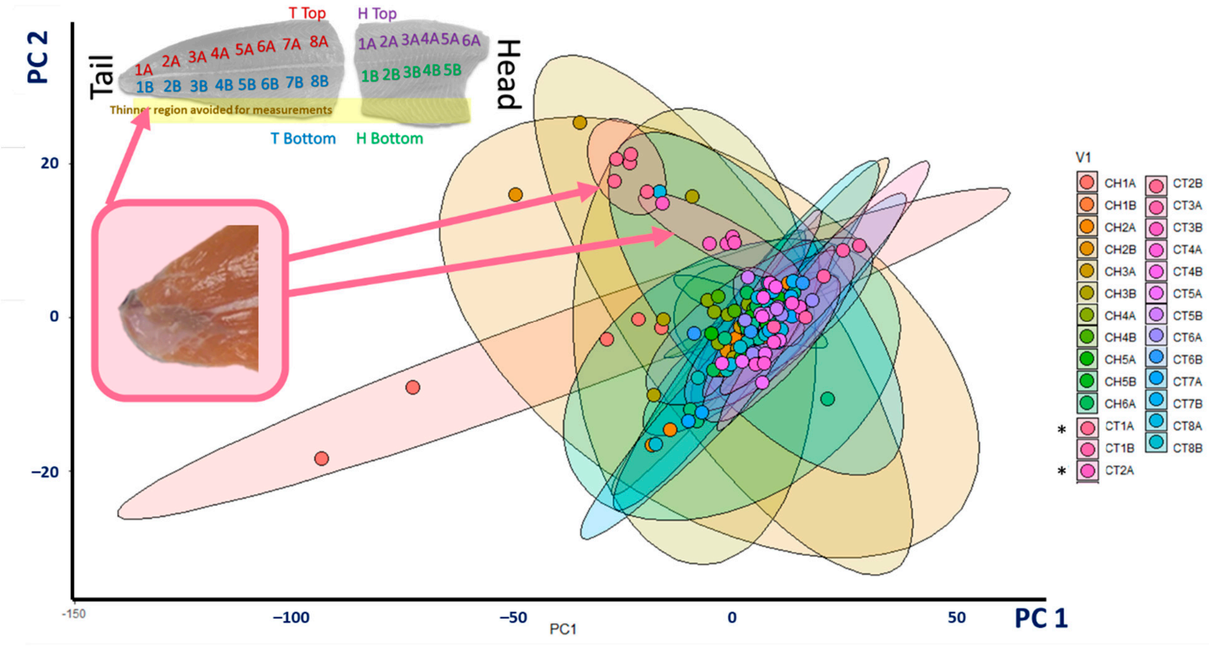

Supplementary Figure S2. Location-specific PCA analysis for calibration salmon fillet (Day 1). INSET: fillet locations and discolouration of Tail section end (pink box). C=Calibration fillet, H=Head, T=Tail. *=regions near fillet periphery that returned highly localised, partially separated confidence ellipses. Confidence ellipses are at 95%.

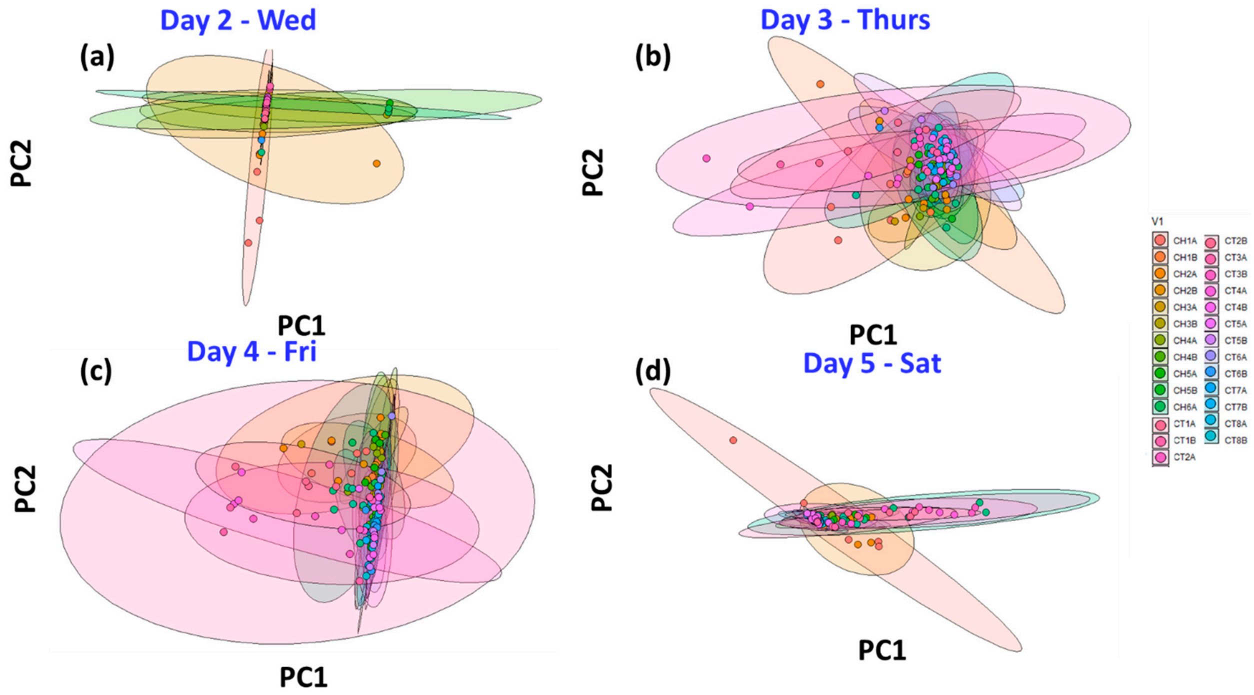

Supplementary Figure S3. Selected PC1 vs. PC2 location-specific PCA plots for Days 2–5. (a) Day 2, (b) Day 3, (c) Day 4, (d) Day 5. 95% confidence ellipses.

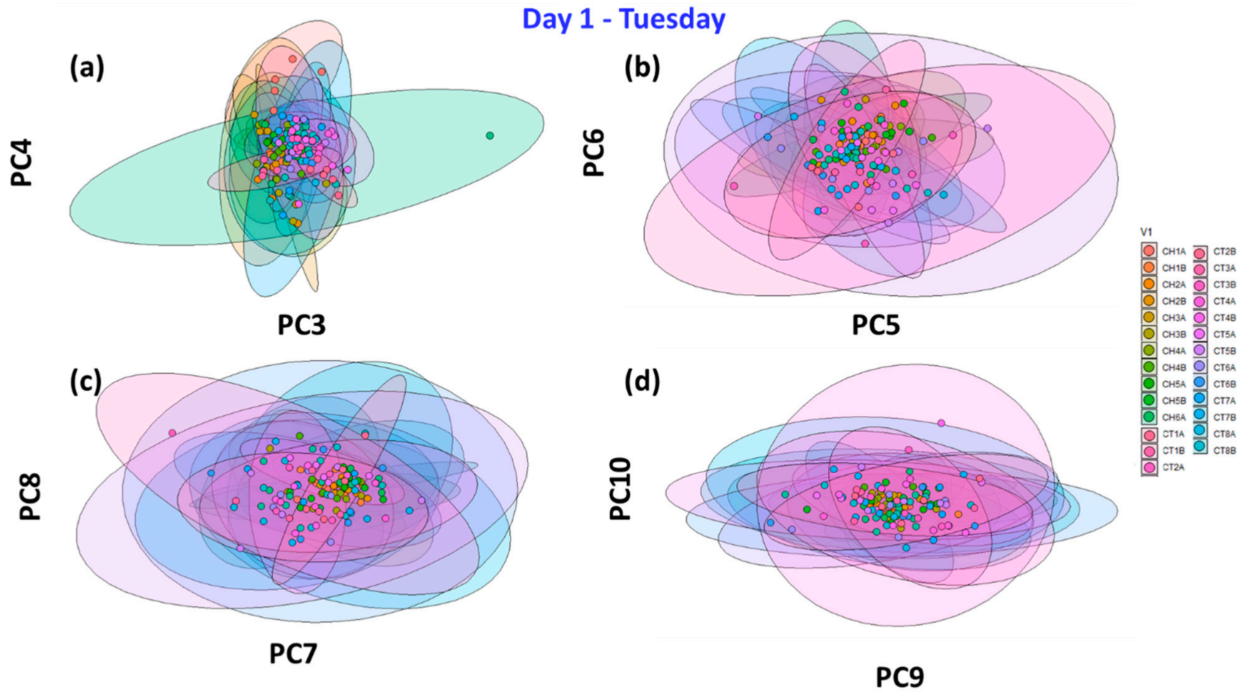

Supplementary Figure S4. Additional location-specific PCA plots for Day 1. (a) PC3 vs. PC4, (b) PC5 vs. PC6, (c) PC7 vs. PC8, (d) PC9 vs. PC10. 95% confidence ellipses.

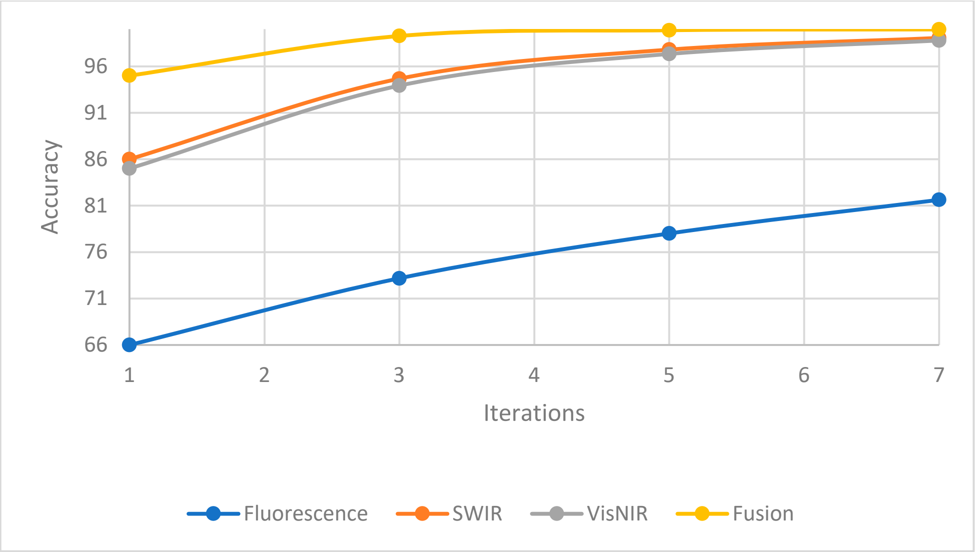

Supplementary Figure S5. Increase in accuracy by measuring multiple points on the test sablefish fillet and how fusion can decrease the number of measurements to achieve the same accuracy level
